# Cell Attachment and Proliferation of Human Adipose-Derived Stem Cells on PLGA/Chitosan Electrospun Nano-Biocomposite 

**DOI:** 10.22074/cellj.2015.4

**Published:** 2015-10-07

**Authors:** Shahnaz Razavi, Saeed Karbasi, Mohammad Morshed, Hamid Zarkesh Esfahani, Mohammad Golozar, Sedigheh Vaezifar

**Affiliations:** 1 Department of Anatomical Sciences and Molecular Biology, School of Medicine, Isfahan University of Medical Sciences, Isfahan, Iran; 2 Department of Medical Physics and Biomedical Engineering, School of Medicine, Isfahan University of Medical Sciences, Isfahan, Iran; 3 Department of Textile Engineering, Isfahan University of Technology, Isfahan, Iran; 4 Department of Biology, Faculty of Sciences, University of Isfahan, Isfahan, Iran; 5 Department of Materials Engineering, Isfahan University of Technology, Isfahan, Iran

**Keywords:** Chitosan, Mesenchymal Stem Cell, PLGA, Scaffold

## Abstract

**Objective:**

In this study, nano-biocomposite composed of poly (lactide-co-glycolide) (PLGA) and chitosan (CS) were electrospun through a single nozzle by dispersing the CS nano-powders in PLGA solution. The cellular behavior of human adipose derived stem cells (h-ADSCs) on random and aligned scaffolds was then evaluated.

**Materials and Methods:**

In this experimental study, the PLGA/CS scaffolds were prepared at the different ratios of 90/10, 80/20, and 70/30 (w/w) %. Morphology, cell adhesion and prolif- eration rate of h-ADSCs on the scaffolds were assessed using scanning electron microscope (SEM), 3-(4, 5-dimethylthiazol-2-yl)-2, 5-diphenyl tetrazolium bromide (MTT) assay and trypan blue staining respectively.

**Results:**

H-ADSCs seeded on the matrices indicated that the PLGA/CS composite matrix with aligned nanofibres and higher content of CS nano-powders gave significantly better performance than others in terms of cell adhesion and proliferation rate (P<0.05).

**Conclusion:**

We found that CS enhanced cell adhesion and proliferation rate, and aligned nanofibers guided cell growth along the longitudinal axis of the nanofibers, which would provide a beneficial approach for tissue engineering.

## Introduction

In recent years, autogenic and allogeneic grafts have been used as substitutes in the treatment of damaged tissues. However, secondary surgery would bring donor site morbidity and could be lead to infection or immune response (1,2). Tissue engineering is a promising approach for repairing or regenerating damaged tissues (3). In this approach, cells should be able to adhere and proliferate in a suitable scaffold in order to regenerate damaged tissues. Viable substitutes can be implanted into the functioning tissue surrounding an injured area (4). For this purpose, biodegradable and biocompatible synthetic or natural polymers have been used to develop scaffolds especially designed to mimic the structure and biological function of the native extracellular matrix (ECM) proteins. An ideal scaffold provides the mechanical support as well as enhances the cell attachment and proliferation rate (5). 

Many researchers have employed the electrospinning technique to fabricate biodegradable and biocompatible scaffolds (6,10). Min et al. (5) fabricated nanostructured matrices of poly (lactide-coglycolide) (PLGA) and PLGA/chitin, composed of PLGA nanofibers and chitin nanoparticles, to examine their effects on cell adhesion and spread of normal human keratinocytes and fibroblasts. They found that the PLGA/chitin composite matrix outperformed the PLGA matrix with regard to cell adhesion and spread of normal human keratinocytes. 

Fabrication of nanofibers from pure Chitosan (CS) is limited by the high repulsive forces between the ionic groups within the polymer backbone arising due to the application of a high electric field during electrospinning (11). 

Previous studies demonstrated that biocomposite of CS have some advantages in terms of mechanical strength, biocompatibility, and antibacterial properties relative to pure CS (12,14). Therefore, we decided to use biocomposite of CS for fabrication of scaffolds. 

In this study, nano-biocomposites composed of PLGA and CS were electrospun through a single nozzle by dispersing the CS nano-powders in PLGA solution. Random (Rd) and aligned-directed (Ad) PLGA and PLGA/CS biocomposites nanofibrous matrices with the different PLGA/CS ratios of 90/10, 80/20 and 70/30 (w/w) % were prepared. The scaffolds produced were subsequently characterized in terms of their water contact angles and mechanical properties. The behavior of human adipose derived stem cells (h-ADSCs) was investigated in terms of cell attachment and morphology as well as proliferation rate of h-ADSCs on the electrospun nano-biocomposite of PLGA and PLGA/CS scaffolds. 

## Materials and Methods

### Fabrication of scaffolds

All reagents, except where specified otherwise, were purchased from Sigma-Aldrich, USA. 

In this experimental study commercially available PLGA granules were dissolved in Trifluroethanol (TFE, Merck, Germany). The block PLGA copolymer solution was loaded in a 1-mL syringe. An electric field (a high-voltage DC power supply of 0-45 kV) was created between the needle used as the anode and the collecting drum used as the cathode. The polymer solution was drawn from the syringe by an accurate infusion control pump (JMS SP 500, Japan). Random PLGA nanofibers and aligned nanofibers were collected on a rotating drum (50 mm in diameter) and wrapped with aluminum foil at 50 rpm and 4000 rpm, respectively. The optimum conditions were obtained with a polymer solution concentration of 24% (w/v) at a feeding rate of 0.25 ml/hours and a high voltage of 13.5 kV. The distance from the collector to the needle tip was 10 cm. The PLGA/CS nano-biocomposites were electrospun onto a rotating collector from a PLGA solution (24% w/v in TFE) containing different weight ratios of 10, 20, and 30 wt% CS nano-powders. These were prepared by the ionic gelation method as described in our previous work (14). The homogenous solution was obtained by slowly adding CS nano-powders and gentle stirring for 12 hours at room temperature. The same optimum electrospinning conditions were obtained as mentioned above. The fabricated scaffolds were dried overnight under vacuum at room temperature. 

### Characterization of scaffolds

Electrospun nanofibrous membranes were sputter-coated with gold, and their morphology was observed using a scanning electron microscope (SEM, Seron Technology AIS 2500, India). The diameters of the resulting nanofibers were determined using the Image J software from the SEM micrographs. The sample thickness was evaluated using cross sections prepared by cryocut (cryocut 1800, reichert, JUNG, Germany) and measured by the Image J software at three points. The measured values were averaged. 

The electrospun samples for transmission electron microscope (TEM) were prepared by collecting them directly onto the copper mesh. A Philips CM12 transmission electron microscope, operated at 120 kV was used to investigate the distribution of nanoparticles.TEM micrographs showed a good distribution of CS nanoparticles in the PLGA fiber. 

To evaluate the effect of CS on the hydrophilicity of electrospun PLGA fiber webs, water contact angles (WCAs) were measured using a contact angle analyzer (Dataphysics, Model, OCA 15 plus, Canada). Measurements were acquired at five independent points and presented as mean ± standard deviation (SD). 

The specimens were carefully cut into 10 mm×50 mm rectangular strips, and their tensile properties were evaluated using a tensile testing machine (Zwick Tensile Tester, 1446, Germany) equipped with a 20-N load-cell. The cross-head speed was 10 mm/minutes. The reported tensile moduli, tensile strengths, and elongations were presented as mean of three tests. 

### Cell isolation and culturing

All procedures were approved by the Ethics Committee of Isfahan University of Medical Sciences. After receiving informed consent human adipose tissue was obtained from the subcutaneous abdominal fat of 3 patients, (21, 34, 40 years old) according to the approved procedure, and cultured as described in our previous study (15). Briefly, samples of adipose tissue were washed extensively with sterile phosphate-buffer saline (PBS) to remove contaminating debris and red blood cells. They were then treated with 0.075% collagenase type I in PBS for 30 minutes at 37˚C. Enzyme activity was neutralized with an equal volume of Dulbecco’s modified eagles medium (DMEM)/ F12 (Gibco, BRL, Paisley, UK) containing 10% fetal bovine serum (FBS). After centrifugation, the cellular suspension was cultured in DMEM/ F12 medium supplemented with 10% FBS and 1% penicillin/streptomycin (Gibco, BRL, Paisley, UK) in 5% CO_2_at 37˚C. When they reached approximately 80% confluency, the cells were detached with 0.25% Trypsin/0.02% Eethylene diamine tetraacetic acid (EDTA, Gibco, BRL, Paisley, UK) at a ratio of 1:3 at every passage. 

Isolated stem cells expressed mesenchymal stem cell-specific markers, including cluster of differentiation (CD) 44 and CD90, but did not express markers for hematopoietic stem cells or endothelial cells, such as CD14, and CD34 (>1%). Thus, isolated stem cells from adipose tissue in this experiment appeared to be mesenchymal stem cells. H-ADSCs used in the present study were from passages 3 to 6. 

The scaffolds were cut out with a punch (14 mm in diameter) and put onto a 24-well culture plate for sterilization using 70% ethanol (Merck, Darmstadt, Germany) and ultraviolet light prior to being placed in the culture medium overnight. The cells were then seeded on the scaffolds at a density of 2×10 ^4^cells/well and incubated in 5% CO _2_at 37˚C. The medium was changed every two days. 

### Cell viability assessment

Cell viability was determined using a 3-(4,5-dimethylthiazol-2-yl)-2,5-diphenyl tetrazolium bromide (MTT) assay after 1, 4, and 7 days. MTT assay is based on the principle that mitochondrial dehydrogenases in viable cells cleave the yellow tetrazolium salt MTT to produce purple formazan crystals. Cells on the scaffolds were incubated with 200 μl of DMEM/F12 and 20 μl of a MTT solution (0.5 mg/ml) in each well for 4 hours at 37˚C under 5% CO _2_until blue precipitation could be seen in cells. The MTT assay media were then discarded. 200 μl of dimethyl sulfoxide (DMSO) was added to extract the MTT formazan and transferred to a 96-well plate where the absorbance of each well was detected by the microplate reader (Hiperion MPR 4+, Germany) at a wavelength of 540 nm (15). 

### Cell adhesion assessment

To count the number of viable cells attached to the scaffolds the substrates were harvested, washed with PBS to remove non-adherent cells, and incubated in 0.5 ml of trypsin at 37˚C for 5 minutes. The trypsinization process was stopped by adding 0.5 ml of DMEM/F12 containing 10% FBS to each sample. After staining with trypan blue, the cell numbers were counted using a hematocytometer with an inverted microscope (Nikon, TS100, Japan). 

### Morphological assessment of cells on scaffolds

The morphology of *in vitro* cultured h-ADSCs on aligned and randomly-oriented PLGA and PLGA/ CS nanofibrous scaffolds was studied. Firstly, the scaffolds with cells remaining after 7 days of cell culture were washed twice with PBS to remove unattached cells which were then fixed for 4 hours using 2.5% glutaraldehyde solution at 4˚C. The scaffolds were then dehydrated in ethanol solution with serial concentrations of 30, 50, 70, 90, and 100% v/v for 15 minutes for each concentration before being air-dried overnight. Dry cellular constructs were finally sputter-coated with gold and observed by SEM. 

### Statistical analysis

Statistical package for social science (SPSS, Chicago, IL, USA) version 18 was used to analyze the data. All the data in this study were presented as means ± SD and analyzed using single-factor ANOVA. The significance level was set at P<0.05.

## Results

### Characterization of scaffolds

The scaffold nomenclature, fiber orientation, PLGA/CS ratio, the average fiber diameter (nm), mechanical properties and water contact angle are presented in table 1. Highly uniform and smooth nanofibers were formed without the occurrence of bead defects in all the random and aligned nanofibrous scaffolds. Using the Image J software of the SEM micrographs, the average fiber diameters of the random and aligned PLGA fibers were determined to be 486 ± 32 nm and 423 ± 30, respectively. No significant differences in diameter were observed for random compared to aligned nanofibers for the respective PLGA and PLGA/CS nanofibers. The nanofiber diameter of PLGA/CS scaffolds decreased and the diameter distribution broadened with increasing CS content. Differences in the diameter by increasing CS content were significant (P<0.05). The presence of CS in the PLGA solution increased conductivity and surface charge densities, which enhanced the whipping instability. Compared with the random nanofibers, the aligned nanofibers were smaller in diameter but no significant differences in the diameter were observed. All the fabricated scaffolds were 70-80 μm in thickness as evaluated by a scanning electron microscope using a cross section prepared by cryocut at three points and measured by Image J software.

Transmission electron micrographs of the PLGA/ CS scaffolds showed that the CS nano-powders were well dispersed on the PLGA nanofibers. The distribution of nanoparticles indicates that the size of the CS nanoparticles on the PLGA/CS scaffolds was smaller than 100 nm. A uniform dispersion of the Cs nanoparticles on the PLGA nanofibrous was obtained with all of the three PLGA/CS ratios (90/10, 80/20, 70/30 w/w %). The WCAs of PLGA/CS scaffolds were measured and compared to that of pure PLGA. The WCA of a PLGA electrospun mat is higher than that of the 1090 Ad and 3070 Ad. The WCA of the PLGA mat is 108.5˚C. In contrast, the WCAs of the 1090 Ad and 3070 Ad decreased to 90.5˚C and 79˚C, respectively, when the CS content was increased. Mechanical properties of both random and aligned electrospun PLGA and PLGA/CS nanofibrous scaffolds are shown in table 1. Clearly, compared with the pure PLGA random scaffold, tensile strength (MPa) and Young’s modulus (MPa) of the random PLGA/CS scaffold was increased with growing CS content.

**Table 1:** Characteristics of the fabricated nanofibrous scaffolds


				Mechanical properties				
Scaffold nomenclature	PLGA/CS	Fiber direction	Fiber diameter (nm)	Young’s modulus (Mpa)	Elongation (%)	Tensile strength (Mpa)	Water contact angle (˚)

100 Rd	100/0	Random	486 ± 32	0.15 ± 0.01	11.0 ± 1.0	0.45 ± 0.04	---
100 Ad	100/0	Align	423 ± 30	0.40 ± 0.03	22.0 ± 1.9	2.60 ± 0.28	108.5
1090 Rd	90/10	Random	393 ± 24*	0.15 ± 0.01	75.0 ± 2.3	1.31 ± 0.10	---
2080 Rd	80/20	Random	364 ± 22*	0.21 ± 0.02	113.0 ± 5.8	1.55 ± 0.15	---
3070 Rd	70/30	Random	320 ± 21*	1.49 ± 0.12	77.0 ± 3.7	3.30 ± 0.32	---
1090 Ad	90/10	Align	352 ± 23*	0.35 ± 0.02	56.0 ± 2.1	4.55 ± 0.54	90.5
2080 Ad	80/20	Align	309 ± 20*	1.76 ± 0.19	57.0 ± 2.2	12.90 ± 1.01	---
3070 Ad	70/30	Align	286 ± 18*	3.00 ± 0.29	36.0 ± 1.8	13.40 ± 1.19	79.0


Data was shown as means ± standard deviation of the mean. Difference mean of diameters of both direction of nanofibers by increasing CS content were significant relative to pure PLGA (*; P<0.05). PLGA; Poly (lactide-co-glycolide), CS; Chitosan, Rd; Random direction and Ad; Aligned direction.

### Cell viability

This study investigated the proliferation rate and adhesion of h-ADSCs onto random and aligned electrospun PLGA and PLGA/CS nanofibrous scaffolds.

**Fig.1:**
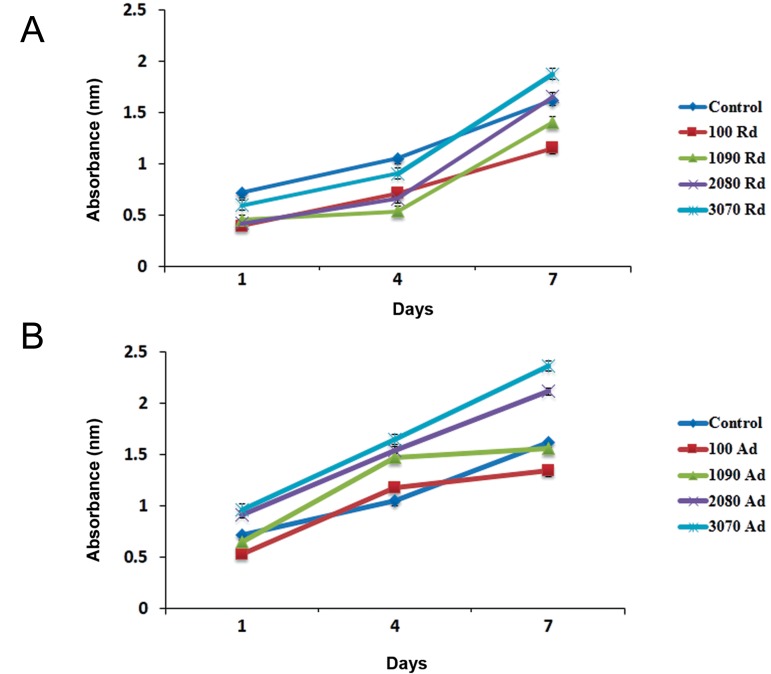
Cell viability on poly (lactide-co-glycolide) (PLGA) and PLGA/chitosan (CS) nanofibrous scaffolds with different weight ratio (90/10, 80/20, 70/30 w/w %) was measured by 3-[4, 5-dimethylthiazol-2-yl]-2, 5-diphenyl tetrazolium bromide (MTT) assay after 1, 4 and 7 days of seeding. Cell viability on random (A), and aligned (B) PLGA and PLGA/CS nanofibrous scaffolds show that cell viability on PLGA/CS scaffolds was significantly increased compared to PLGA scaffolds (P<0.05). Moreover, cell viability was significantly increased in the aligned nanofibrous scaffolds (Ad) as compared to the random nanofibrous scaffolds (Rd) after 7 days of culture (P<0.05).

Cell viability on the PLGA and PLGA/CS nanofibrous scaffolds with different weight ratio (90/10, 80/20, 70/30 w/w %) was measured by MTT assay after 1, 4 and 7 days of seeding (Fig .1). H-ADSCs were cultured on a T75 flask as the control group. Cell viability on random (Fig .1A), and aligned (Fig .1B). PLGA and PLGA/CS nanofibrous scaffolds, show that cell viability on PLGA/ CS scaffolds significantly increased compared to the case of PLGA scaffolds (P<0.05). Moreover, cell viability was significantly increased in the aligned nanofibrous scaffolds as compared to the random nanofibrous scaffolds after 7 days of culture (P<0.05).

### Cell adhesion

**Fig.2:**
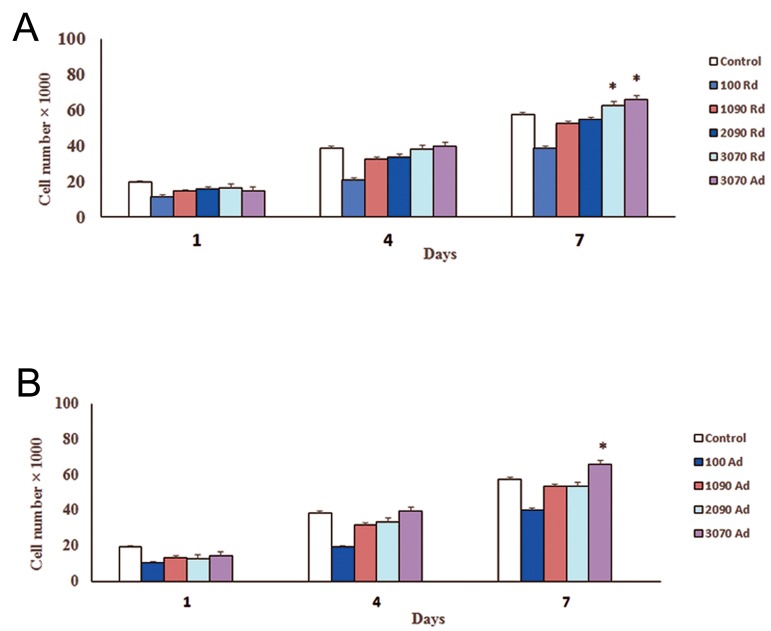
Cell adhesion onto the random (Rd), and aligned (Ad) poly (lactide-co-glycolide) (PLGA) and PLGA/chitosan (CS) nanofibrous scaffolds with different weight ratio (90/10, 80/20, 70/30 w/w %) after 1, 4, and 7 days. Cell adhesion and cell number on the PLGA/CS membrane were increased with increasing CS content in the scaffolds. In addition, cell adhesion on PLGA/CS random nanofibrous scaffolds (A) was less than the aligned PLGA/CS nanofibrous scaffolds (B). After 7 days of seeding, a significant mean difference was observed in the cell number on nanofibrous scaffolds with a high CS content as compared to day 1 on both electrospun scaffolds (*; P<0.05).

Figure 2 shows the cell adhesion onto the PLGA and PLGA/Cs scaffolds with random nanofibrous scaffold was measured by cell counts after 1, 4, and 7 days of seeding (Fig .2A). As shown, cell adhesion on PLGA/CS membrane was increased with increasing CS content in the scaffold. 

In addition, cell adhesion on the aligned PLGA/ CS nanofibrous scaffolds was more than the random PLGA/CS nanofibrous scaffolds (Fig .2B). After 7 days of seeding, a significant mean difference was observed in the cell numbers for nanofibrous scaffold with high CS content, compared to day 1, on both electrospun scaffolds (P<0.05). 

### Cell morphology

**Fig.3:**
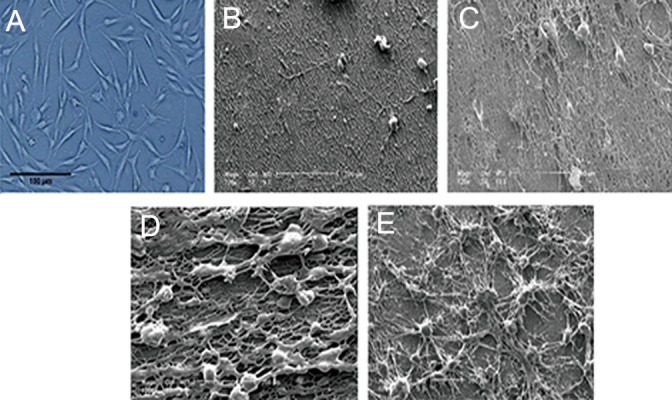
A. Photomicrograph from inverted microscope in control group, and scanning electron microscope (SEM) micrographs of human adipose derived stem cells (h-ADSCs) cultured for 7 days on poly (lactide-co-glycolide) (PLGA) and PLGA/chitosan (CS) nanofibrous scaffolds with different weight ratios: B. 100 Ad, C. 1090Ad, D. 3070 Ad and E. 3070 Rd.

Figure 3 presents the morphologies of h-ADSCs cultured for 7 days on 100 Ad, 1090 Ad, 3070 Ad, and 3070 Rd scaffolds. It can be seen that h-ADSCs adhered well on the surface of all the electrospun scaffolds, and further that the direction of cell growth on the aligned scaffolds was partially parallel to that of fiber alignment, whereas it was random on the random scaffolds. Cell morphology exhibits a round shape on the random scaffolds but a partly spindle-like shape on the aligned scaffolds. The number of cells attached onto the scaffold increased with increasing CS content in the biocomposite scaffold. HADSCs spread across the surface of the PLGA/ CS composites, while those on the pure PLGA mat were sporadically distributed.

## Discussion

In previous work, both random and aligned PLGA and PLGA/CS scaffolds have been fabricated using two different methods by electrospinning (16). 

In the current study scaffolds fabricated by dispersing CS nano-powders in a PLGA solution using the single nozzle electrospinning method were investigated for cellular behavior, cell adhesion and proliferation of h-ADSCs. 

In general, the electrospun scaffold may provide a favorable matrix for cell adhesion and proliferation because it physically mimics the ECM structure of native tissues, such that the morphology of the fibers is very similar to that of native human ECM (17). Therefore, the electrospun PLGA/CS composite membranes could mimic the natural ECM and positively promote cell-matrix and cellcell interactions. 

Compared to random nanofibers, the aligned nanofibers were smaller in diameter due to the drawing effect induced by the rotating drum as the jet made contact with the surface of the drum (18). 

CS is a variety of natural cationic polyelectrolyte. The presence of CS nano-powders in the PLGA solution increased conductivity and surface charge densities, which enhanced the whipping instability. The WCA results indicate that the CS in the biocomposites gave rise to hydrophilicity. This would not only prevent the loss of bodily fluids and nutrients in the *in vivo* tests, but would also improve the relatively low cell attachment and proliferation rate of pure PLGA membrane (16). 

Additionally, the hydrophilicity of a material contributes significantly to the cell attachment and proliferation rate (19), so increasing the Cs content in the biocomposite scaffold increases the number of cells attached to the scaffold. It is well known that cell adhesion may be affected by the surface hydrophilicity of the scaffold, and that a hydrophilic surface leads to improved cell adhesion compared to a hydrophobic surface (20). 

In the present study, the method of preparation of the scaffold is different from that used in other works. We used a single nozzle electrospinning method for dispersing the CS nano-powders across the PLGA nanofibrous scaffold and found that an aligned nanofibrous scaffold with a high percentage of CS nano-particles (3070 Ad) provided a beneficial approach for cell adhesion and proliferation of ADSCs. 

However, in the other researches preparation method and application of scaffold were altered (21,24). 

Wang et al. (21) found that cell attachment and proliferation rate of h-ADSCs were promoted on a porous scaffold composed PLGA/CS *in vitro* and *in vivo*. It has been shown that Cs scaffold synthesized by freeze-drying and containing PLGA nanoparticles can act as an anticancer drug and can prevent tumor formation *in vitro* conditions (22). 

In addition, Cs film containing PLGA nanoparticles can be used for restricted drug release (23). While, Nazemi et al. (24) have prepared Cs/58Sbioactive glass (58S-BG) scaffold containing PLGA nanoparticles using the sol-gel method. They suggested this nano-composite could be considered in bone tissue engineering scaffolds. 

Min et al. (5) fabricated matrices of PLGA and PLGA/chitin, composed of PLGA nanofibers and chitin nanoparticles, to examine the effects of PLGA and PLGA/chitin matrices on cell adhesion and spread of normal human keratinocytes and fibroblasts. They found that the PLGA/chitin composite matrix may be a better candidate than the PLGA matrix in terms of cell adhesion and spread of normal human keratinocytes. 

Hong and Kim (25) described two processing methods for the fabrication of polycaprolactone (PCL)/CS biocomposites. In the first method, CS was used to reinforce electrospun PCL nanofibers by a co-electrospinning process in which a mixture of PCL and CS powders was electrospun in a single step. In the second method, CS was deposited on electrospun PCL fibers by an air-spraying process. They found that the biological response of mesenchymal stem cells on the biocomposites was superior to that on pure PCL in terms of improved cell attachment and higher proliferation rate. 

Meng et al. (3) produced random and aligned PLGA and PLGA/gelatin biocomposite scaffolds by electrospinning. They reported that this biocomposite can improve the hydrophilicity of scaffold, however the diameter of the fibers and the mechanical properties of the scaffolds were reduced. The orientation of osteoblast processes were parallel to the direction of fibers in the aligned nanofibrous scaffold, but the cell number was similar in both two scaffolds. While, in consistent with recent work (26), we found that direction of nanofibers can support cell growth along the longitudinal axis of the nanofibers. 

Prabhakaran et al. (27) studied the potential of neurogenic differentiation of human bone marrow derived mesenchymal stem cells on poly(L-lactic acid)-co-poly-(3-caprolactone)/Collagen (PLCL/ Coll) electrospun nanofibrous scaffold. They found that the cell number was higher on PLCL/ Coll nanofibrous scaffold relative to those grown on PLCL. These results are in accordance with our observation that Cs-supplemented biocomposites would make excellent materials for tissue engineering applications. 

Recently, Wang et al. (28) show that the presence of nano-hydroxyapatite in CS/PLGA scaffold can enhance proliferation and osteogenic differentiation of human umbilical cord mesenchymal stem cells. 

## Conclusion

Overall, we found that using CS nanoparticles to fabricate PLGA/CS composite enhances the hydrophilicity and the mechanical properties of the scaffolds. Cell adhesion and proliferation increase with increasing CS content and aligned nanofibers, indicating that the aligned nanofibrous scaffold can provide a beneficial approach to tissue regeneration.
